# Transcriptomic analysis of the effect of remote ischaemic conditioning in an animal model of necrotising enterocolitis

**DOI:** 10.1038/s41598-024-61482-9

**Published:** 2024-05-11

**Authors:** Ian Howard Jones, Jane Elizabeth Collins, Nigel John Hall, Ashley Ivan Heinson

**Affiliations:** 1 University Surgery Unit, Faculty of Medicine, University of Southampton, Tremona Road, Southampton, UK; 2 Birmingham Children’s Hospital, Steelhouse Lane, Birmingham, UK; 3 Southampton Children’s Hospital, Tremona Road, Southampton, UK; 4 Clinical and Experimental Sciences, University of Southampton School of Medicine, Southampton, UK; 5 Clinical Informatics Research Unit, Cancer Sciences, University of Southampton School of Medicine, Southampton, UK

**Keywords:** Mechanisms of disease, Cell biology, Computational biology and bioinformatics, Transcriptomics, Inflammation, Gastrointestinal diseases, Gastrointestinal models

## Abstract

Necrotising enterocolitis (NEC) has a complex pathophysiology but the common end-point is ischaemia reperfusion injury (IRI) and intestinal necrosis. We have previously reported that RIC significantly reduces the intestinal injury in a rat model of NEC. Here we describe the changes in intestinal mRNA occurring in the intestine of animals exposed to IRI, both with and without RIC. Related rat-pups were randomly assigned to four groups: SHAM, IRI only, RIC only and RIC + IRI. IRI animals, underwent 40 min of intestinal ischaemia, and 90 min of reperfusion. Animals that underwent RIC had three cycles of 5 min of alternating ischaemia/reperfusion by means of a ligature applied to the hind limb. Samples from the terminal ileum were immediately stored in RNA-preserving media for later next generation sequencing and transciptome analysis using R v 3.6.1. Differential expression testing showed that 868 genes differentially expressed in animals exposed to RIC alone compared to SHAM and 135 in the IRI and RIC group compared to IRI alone. Comparison between these two sets showed that 25 genes were differentially expressed in both groups. Pro-inflammatory molecules: NF-ĸβ2, Cxcl1, SOD2 and Map3k8 all show reduced expression in response to RIC. Targeted gene analysis revealed increased expression in PI3K which is part of the so-called RISK-pathway which is a key part of the protective mechanisms of RIC in the heart. Overall, this transcriptomic analysis shows that RIC provides a protective effect to the intestine via anti-inflammatory pathways. This could be particularly relevant to treating and preventing NEC.

## Introduction

Necrotising enterocolitis (NEC) is a devastating disease with a high mortality and morbidity for the survivors^1^ The pathophysiology of NEC is complex, but ischaemia–reperfusion injury (IRI) is a common end-point of different, overlapping pathophysiological processes^2^.

Remote ischaemic conditioning (RIC) is an endogenous phenomenon whereby the application of short cycles of non-injurious ischaemia and reperfusion to one organ system (i.e. skeletal muscle) provides a systemic protective effect against ischaemia–reperfusion injury^3^. We have previously reported the protective effect of remote ischaemic pre-conditioning (pre-RIC) in an animal model based on ischaemia–reperfusion injury to the intestine in a rat pup^4^. In this model, the application of pre-RIC resulted in a dramatic reduction in intestinal injury. Tissue harvested from the same animals that was stored in RNA-preserving media was used for this study.

Koike et al*.* (2020)^5^ used a neonatal mouse gavage model of NEC and showed that RIC is protective against intestinal injury and that improvement of the intestinal microcirculation is key to the protection. Similarly, it has been shown that RIC reduces several pro-inflammatory cytokines that are reaised in NEC including GATA3, IFNγ, IL1β, IL6, IL17, IL22, and TNFα.

Whole transcriptome analysis of messenger Ribonucleic Acid (mRNA) is a powerful tool for studying complex biochemical pathways. In recent years, a few papers have studied the changes in gene expression triggered by remote ischaemic conditioning (RIC).

Yoon et al*.* (2015)^6^ used microarray analysis to study the gene expression changes in a porcine model of preRIC and renal Ischaemia reperfusion injury (IRI). They found that RIC had effects on the expression of multiple cytokines (including Interleukin-10 (IL-10) and Transforming Growth Factor beta (TGF-β), as well as modulating the complement and coagulation cascades. However, there are potentially significant differences in their experimental design. Firstly, in the model of renal IRI, RIC did not alter the primary end-point, which was a surrogate for renal function. Secondly the tissues were harvested two days after the IRI and thus do not capture the immediate changes in gene-expression.

The analysis of gene expression in a porcine model of myocardial infarction with post-ischaemic conditioning was reported by Lukovic et al*.* in 2019^7^. Unsurprisingly, many of the gene expression changes seen in the myocardium are the same in animals exposed to post conditioning as those who were not; both groups undergoing a myocardial injury. However, there were distinct genes whose regulation was different in animals who underwent conditioning. They concluded that ischaemic conditioning downregulates Extra Cellular Matrix (ECM)-proteinases, ribosomal subunits and platelet and leukocyte adhesion molecules. Additionally, post-conditioning inhibited the activation of inflammatory leukocytes.

More recently, Zou et al. used transcriptomic analysis on rat myocardium and identified that increased expression of ADAMTS15 may be important in the mechanisms of RIC leading to cardioprotection by reducing inflammatory changes^8^.

Here we report changes in expression patterns in intestinal tissue. The majority of research into RIC has focused on the heart and to a lesser extent the brain. Therefore the processes within intestinal tissue which are important in the context of NEC, are not well understood and is the focus of our studies. There has already been a phase I trial on the use of RIC in human infants and the protocol for a phase II^9^, international study has been published. There is a direct pathway for translation to clinical practice.

## Results

### RNA quality

The RNA Integrity Number (RIN) scores for each sample are shown in Table 1: 23 out of the 24 samples had RIN scores greater than 8. The Concentration of RNA in each sample ranged from 1620 to 5060 ng/µl.

**Table 1:** Quality of RNA in each sample.

Sample	Concentration of RNA (ng/µl)	RIN
1	1990	8.7
2	2460	9.4
3	2940	8.3
4	2540	9.2
5	3520	8.1
6	2920	9.1
7	2760	9.5
8	3780	9.0
9	2220	9.4
10	2760	9.5
11	3360	7.4
12	2780	9.3
13	2800	8.1
14	2980	9.5
15	2220	9.5
16	5060	8.2
17	4500	8.9
18	4540	8.2
19	3600	9.5
20	1620	9.3
21	2860	9.7
22	3900	9.1
23	2820	10.0
24	2800	8.9

This table shows the quality of RNA from each sample that underwent sequencing. Data provided by Qiagen. RIN > 8 indicates high quality sample^47,48^.

*RIN* RNA Integrity Number.

### Data exploration and quality control

Before proceeding to analyse the differential gene expression, we excluded the lowly expressed mRNAs and then used hierarchical clustering, and Principal Component Analysis (PCA) to ensure we had no outliers in the dataset indicating a technical issue with the Ribonucleic Acid (RNA) processing and the Next Generation Sequencing (NGS). Figure S1 shows box plots of the raw sample counts of mRNA hits and the same dataset filtered to exclude lowly expressed mRNAs. This filtering reduces the noise from lowly expressed mRNA and thus the need for statistical correction of multiple testing. Hence this reduces the risk of a Type II statistical error. These charts also show very similar mRNA counts across all samples.

The hierarchical clustering (using Euclidean distance and Average linkage) showed that in the whole dataset the IRI vs SHAM dominates over RIC vs SHAM in the patterns of gene expression, with samples from IRI and RIC + IRI clustering together and SHAM and RIC + SHAM clustering together (Fig.S2). The PCA was labelled by known phenotypical factors (Age in days, gender, weight, litter, and date of procedure) there was no clustering by any of these specific factors. Sample 3 was a potential outlier in the PCA but not within raw counts/IQR plot. On the basis of these analyses, no samples needed to be excluded from further analysis of differential gene expression. (Figs. S3 and S4). Similarly we did not find any confounding variables or batch effects that would adversely affect the analysis.

### Differential expression testing

**Figure 1:**
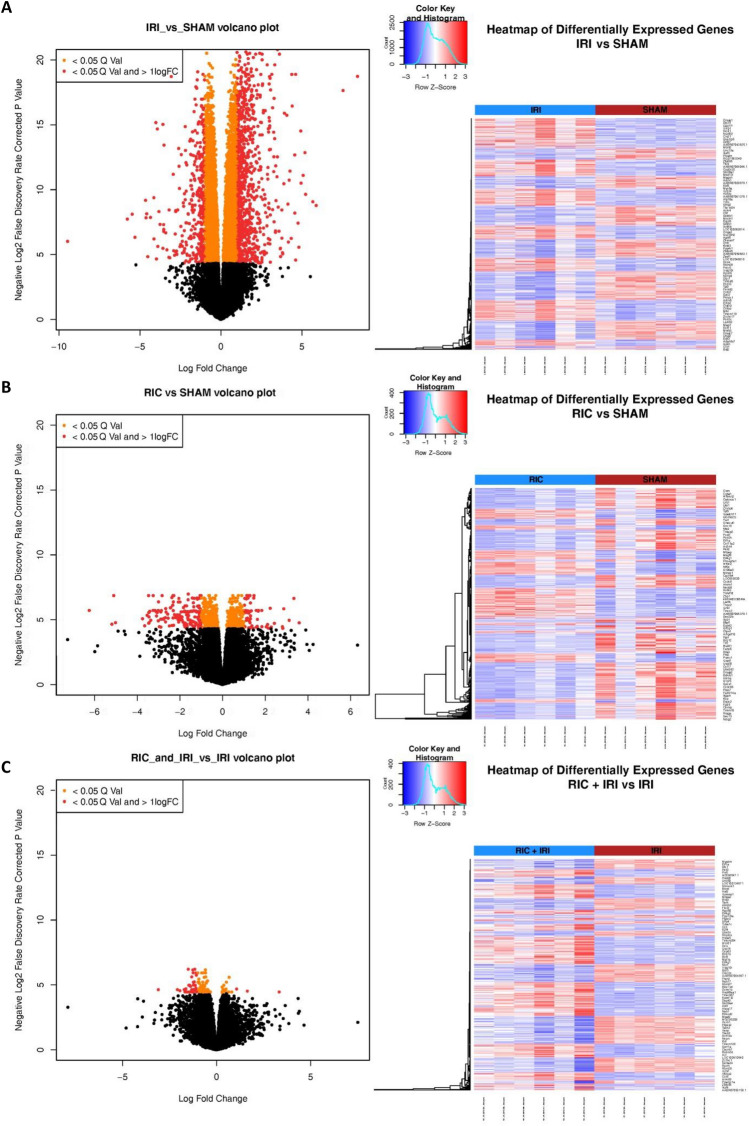
The differential expression results as identified by edgeR for RNA sequencing experiments on rat pups of different experimental groups. The volcano plots are coloured based on associated adjusted *p* value output of the edgeR differential expression testing method and the log fold change of the expression change. Orange signifies a result for the gene that is < 0.05 Q value. Red signifies a result for < 0.05 q value and > 1 log fold change. The heatmap represent the genes identified to be differentially expressed between the two groups (Q value < 0.05 when output from edgeR). The heatmaps are groups by condition. (**A**) Shows the differential expression results for rat pups exposed to Ischemia reperfusion injury vs SHAM surgery. (**B**) Shows the differential expression results for rat pups exposed to Remote ischaemic conditioning vs SHAM surgery. (**C**) Shows the differential expression results for rat pups exposed to Remote ischaemic injury and Ischaemic reperfusion injury vs Ischemia reperfusion injury. *RIC* Remote ischaemic conditioning; *SHAM* SHAM surgery, *IRI* Ischaemia reperfusion injury.

**Figure 2:**
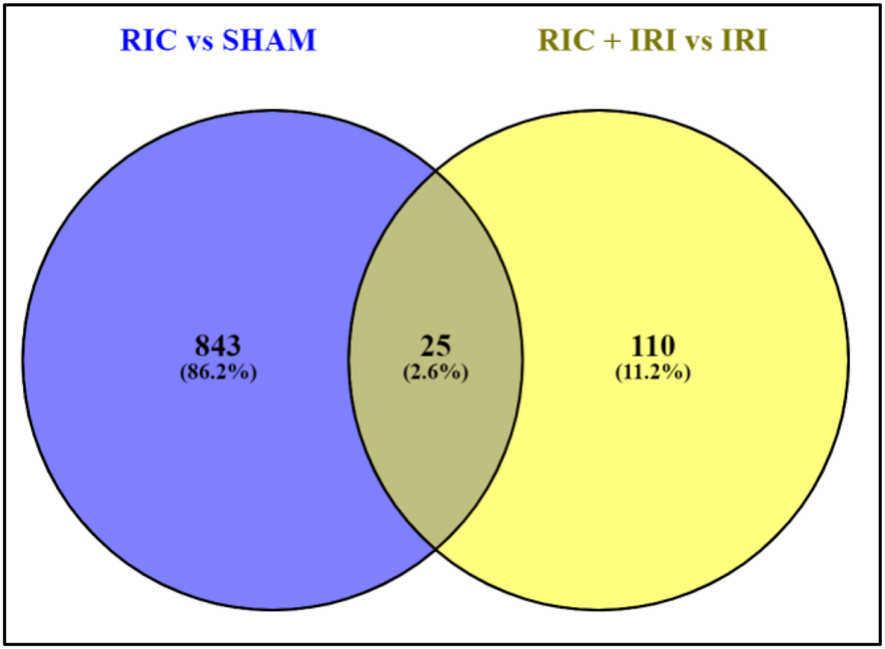
Venn diagram visualising the overlap of statistically significant genes between the experimental group comparisons of remote ischaemic conditioning vs SHAM surgery and remote ischaemic conditioning plus ischaemia reperfusion injury vs ischemia reperfusion injury. Statistically significant genes are defined by an adjusted *p* value (Q value) < 0.05 utilising the standard methodology for differential expression testing using edgeR. *IRI* Ischaemic reperfusion injury; *RIC* remote ischaemic conditioning; *SHAM* Sham surgery.

We report three different two group comparisons: IRI vs SHAM; RIC + IRI + IRI; and RIC vs SHAM. The full tables of differentially expressed genes are included in the additional material. In animals that underwent IRI compared to SHAM 6772 genes showed statistically significant differential expression (q value < 0.05) (Fig. 1). In animals that underwent RIC only (compared to SHAM) there were 868 genes that showed differential expression (Fig. 1) and the comparison between RIC + IRI and IRI along showed 136 genes with statistically significant gene expression (Fig. 1). Figure 2 shows the overlap in genes that showed differential expression in both the RIC vs SHAM and RIC + IRI vs IRI groups.

Among these 25 genes found in both bilateral comparisons (Table 2), are several genes which are likely candidates for involvement in a putative RIC pathway within the intestine as they are known to be important in inflammatory pathways^10^. These include CXCL1 which is an important chemoattractant, TIMP1 which is anti-apoptosis, CD-55 which is known to be a complement regulator, SOD2 which is involved in the detoxification of reactive oxygen species and Nfκβ2 which is a key part of the innate inflammation pathway^10^.

**Table 2:** Gene Symbols identified as differentially expressed in both of the comparisons remote ischaemic conditioning vs SHAM surgery and remote ischaemic conditioning plus ischaemia reperfusion injury vs ischaemic reperfusion injury.

1	Lrrc8c	10	Malsu1	18	Map3k8
2	Ugdh	11	Spry2	19	Sod2
3	Cxcl1	12	Timp1	20	Nfkb2
4	Adamts4	13	Tfpi2	21	Procr
5	Cd55	14	Plau	22	Usp36
6	Trib1	15	Coq10b	23	Kcne4
7	Adamts8	16	S1pr3	24	Gstm5
8	Pde4b	17	Ifitm3	25	AABR07047256.1
9	RGD1563365				

Deferentially expressed genes were identified utilising edgeR standard methods. Genes with an adjusted *p* value (Q value) < 0.05 were deemed to be significant.

*SHAM* Underwent fake surgery; *RIC* Remote ischaemic conditioning; *IRI* Ischaemic reperfusion injury; *IRI* + *RIC* Ischaemic reperfusion injury plus remote ischaemic conditioning; *NEC* Necrotising enterocolitis.

### Differential expression: targeted gene analysis

**Figure 3:**
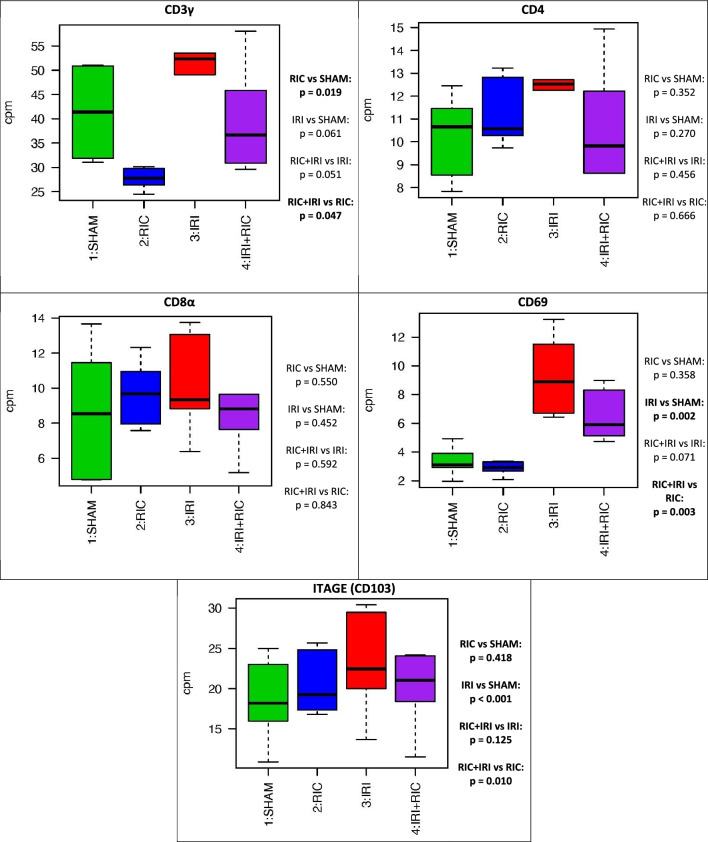
The expression differences in specific cell type markers for T-cells genes across the four experimental conditions. T tests were used to generate *p*-values across the groups. *SHAM* Underwent Sham surgery; *RIC* Remote ischaemic conditioning; *IRI* Ischaemic reperfusion injury; *IRI* + *RIC* Ischaemic reperfusion injury plus remote ischaemic conditioning.

Table 3 shows the results of the targeted gene analysis. In terms of cell markers, RIC significantly reduced the expression of macrophage markers (Fig. S5). CD3 was reduced by RIC suggesting a reduction in T-cells but both CD4 and CD8a were not statistically significantly changed (Fig. 3). Both fibroblast and alpha-smooth muscle markers were increased by IRI and this effect was mitigated by RIC (Fig. S6).

**Table 3:** Four way comparison of gene expression of specific markers: a) Cell type markers; b) Molecular pathways: Hypoxia, cell toxicity and cell repair c) Molecular pathways known to be involved in NEC pathophysiology and d) Molecules that form the RISK pathway in cardiac ischaemic conditioning.

	Marker used	Result	*p*-value	Figure
Cell type				
Immune cells	PTPRC (CD45)	No Change		S5
Macrophages	CD163	Increased by RIC	RIC vs SHAM: 0.003/RIC + IRI vs IRI: < 0.001	
	CD164	No Change		
	MRC1 (CD206)	Reduced by RIC AND IRI combined	RIC + IRI vs RIC: 0.006	
	CD68	No Change		
	ITGAX	Reduced by RIC	RIC vs SHAM: 0.014/RIC + IRI vs IRI < 0.001	
B-cells	MS4A1 (CD20)	No Change		
	CD19	No Change		
T-cells	CD3γ	Reduced by RIC	RIC vs SHAM: 0.019	3
	CD4	No Change		
	CD8α	No Change		
	CD69	Increased by IRI	IRI vs SHAM: 0.002/RIC + IRI vs RIC: 0.003	
	ITGAE (CD103)	No Change		
Natural Killer Cells	NCAM1 (CD56)	Increased by RIC, Increased by IRI	RIC vs SHAM: < 0.001/IRI vs SHAM: 0.029	
	KIR3DL1	No Change		
Endothelial cells	PECAM1 (CD109)	No Change		
Neutrophils	FUT4 (CD15)	Reduced by IRI	IRI vs SHAM: < 0.001/RIC + IRI vs RIC: 0.003	
		(Effect mitigated by RIC)	RIC + IRI vs IRI: 0.043	
Fibroblasts	VIM	Increased by IRI	IRI vs SHAM: < 0.001/RIC + IRI vs RIC: 0.025	S6
		(Effect mitigated by RIC)	RIC + IRI vs IRI: 0.022	
	CDH2	No Change		
	ACTA2	Increased by IRI	RIC vs SHAM: 0.001	
		Reduced by RIC	IRI vs SHAM: 0.02/RIC + IRI vs RIC: 0.043	
Alpha Smooth Muscle	PAFAH1B3	Reduced by IRI	IRI vs SHAM: 0.049	S6
	DES	Increased by IRI	IRI vs SHAM: < 0.001/RIC + IRI vs RIC: 0.013	
		Reduced by RIC	RIC vs SHAM: 0.01/RIC + IRI vs IRI: 0.013	
	CDH2	No Change		
Biological processes				
Hypoxia	HIF-1a	Increased by IRI	IRI vs SHAM: < 0.001/RIC + IRI vs RIC: < 0.001	4
		(Effect mitigated by RIC)	RIC + IRI vs IRI: 0.005	
	EPAS1 (HIF-1β)	No Change		
	ARNT (HIF-2α)	Increased by IRI	IRI vs SHAM: 0.009/IRI + RIC vs RIC: < 0.001	
		Reduced by RIC	RIC vs SHAM: 0.038/RIC + IRI vs IRI: 0.023	
	VEGFA	Increased by IRI	IRI vs SHAM: < 0.001/RIC + IRI vs RIC: < 0.001	
		(Effect mitigated by RIC)	RIC + IRI vs IRI: 0.041	
Cell Toxicity	GZMA	No Change		
	GZMB	No Change		
Cell Repair	KRT-7	Increased by IRI	IRI vs SHAM: 0.005/RIC + IRI vs RIC: 0.01	S7
		(Effect mitigated by RIC)	RIC + IRI vs IRI: 0.04	
	KRT-8	Increased by IRI	IRI vs SHAM: 0.012/IRI + RIC vs RIC: 0.02	
	KRT-18	Increased by IRI	IRI vs SHAM: 0.001/RIC + IRI vs RIC: 0.004	
		(Effect mitigated by RIC)	RIC + IRI vs IRI: 0.003	
	KRT-19	Increased by IRI	IRI vs SHAM: 0.018/RIC + IRI vs RIC: 0.016	
Specific biological pathways				
Molecules known to be involved in NEC pathophysiology	TLR-2	Increased by IRI	IRI vs SHAM: 0.044/RIC + IRI vs RIC: 0.005	5
		Reduced by RIC	RIC vs SHAM: 0.042/RIC + IRI vs IRI: 0.005	
	TLR-4	Decreased by IRI	IRI vs SHAM: 0.005/RIC + IRI vs RIC: 0.005	
	MyD88	Increased by IRI	IRI vs SHAM: 0.002/RIC + IRI vs RIC: 0.001	
	NF-KB1	Increased by IRI	IRI vs SHAM: 0.006/RIC + IRI vs RIC: 0.003	
		Reduced by RIC	RIC vs SHAM: 0.032/RIC + IRI vs IRI: 0.019	
	CD17	No Change		
	iFABP	No Change		
Molecules known to be involved in RIC in cardiac tissue	MEK1	Reduced by IRI	IRI vs SHAM: 0.002/RIC + IRI vs RIC: 0.032	6
	MEK2	Reduced by IRI	IRI vs SHAM: < 0.001	
		Reduced by RIC	RIC vs SHAM: 0.009	
	PI3K	Reduced by IRI	IRI vs SHAM: < 0.001/RIC + IRI vs RIC: < 0.001	
		Increased by RIC	RIC vs SHAM: 0.023	
	P38	Reduced by IRI	IRI vs SHAM: < 0.001/RIC + IRI vs RIC: 0.012	
	PKC	Reduced by IRI	IRI vs SHAM: < 0.001/RIC + IRI vs RIC: 0.01

**Figure 4:**
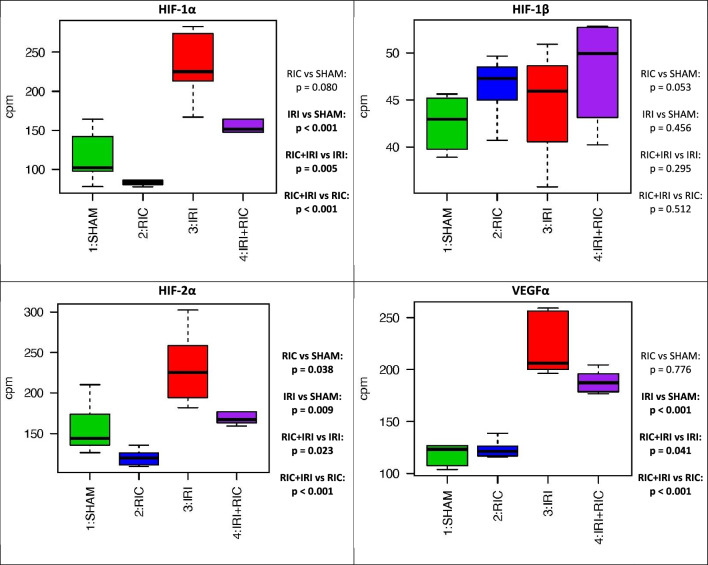
The expression differences in specific cell type markers for hypoxic pathway genes across the four experimental conditions. T tests were used to generate *p*-values across the groups. *SHAM* Underwent Sham surgery, *RIC* Remote ischaemic conditioning; *IRI* Ischaemic reperfusion injury; *IRI* + *RIC* Ischaemic reperfusion injury plus remote ischaemic conditioning.

HIF1-α, HIF2-α and VEGFα—markers of hypoxia—were all statistically significantly increased by IRI and reduced by RIC (Fig. 4). Similarly, markers of cell-repair mechanisms (KRT-7,-8,-18 and -19) were increased in animals exposed to IRI with this effect mitigated by RIC (Fig. S7).

**Figure 5:**
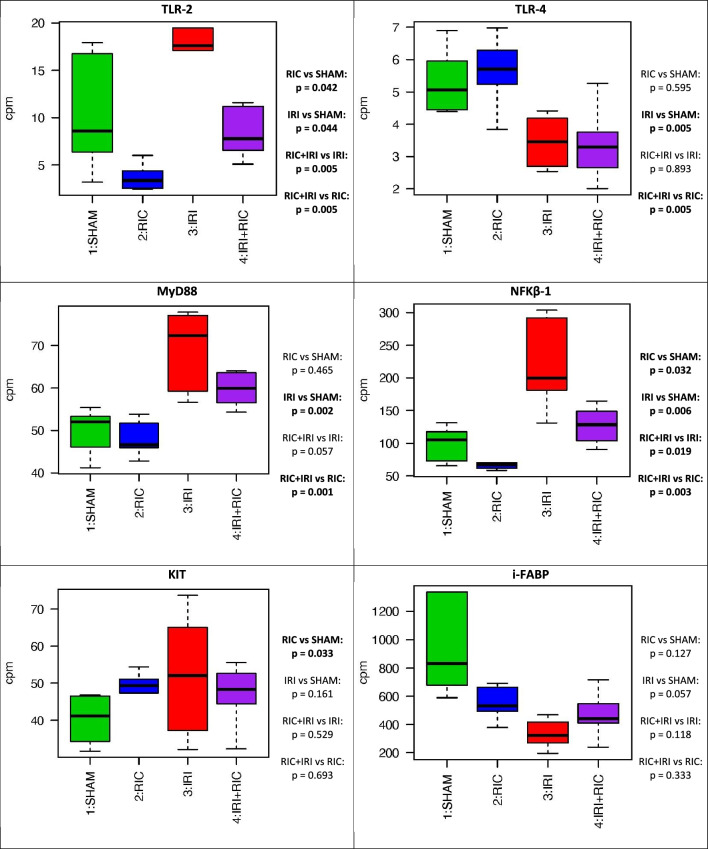
The expression differences in specific cell type markers for molecules known to be involved in necrotising enterocolitis genes across the four experimental conditions. T tests were used to generate *p*-values across the groups. *SHAM* Underwent Sham surgery; *RIC* Remote ischaemic conditioning; *IRI* Ischaemic reperfusion injury; *IRI* + *RIC* Ischaemic reperfusion injury plus remote ischaemic conditioning; *NEC* Necrotising enterocolitis.

**Figure 6:**
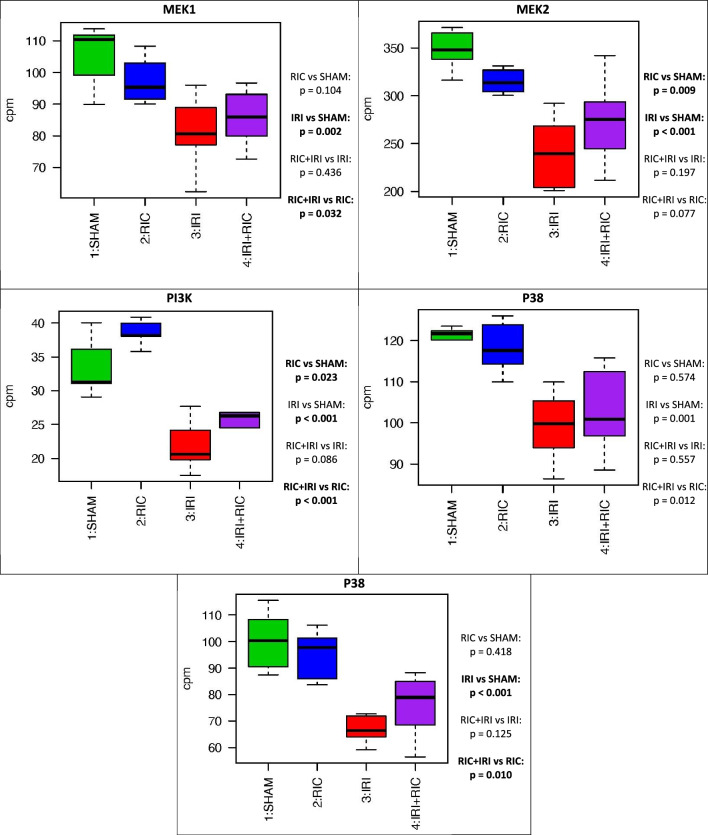
The expression differences in specific cell type markers for the RISK pathway genes across the four experimental conditions. T tests were used to generate *p*-values across the groups. *SHAM* Underwent Sham surgery; *RIC* Remote ischaemic conditioning; *IRI* Ischaemic reperfusion injury; *IRI* + *RIC* Ischaemic reperfusion injury plus remote ischaemic conditioning.

Toll-like Receptor-2, Toll like receptor-4, MyD88 and Nfκβ1 were all increased by IRI. This effect was mitigated by RIC (Fig. 5). MEK1, MEK2, P48 and PKC were all reduced by IRI, suggesting a role in ischaemic injury. PI3K in increased with RIC. (Fig. 6).

## Discussion

The protective effect of RIC is well-established in multiple species and multiple organ systems^3^. In this work we showed a profound reduction in intestinal injury in rat pups exposed to IRI^4^. The mechanisms by which RIC confers this protective effect are complex and significant work over the past decades has elucidated multiple potential pathways involved. Within the context of the intestine, the 'end-organ' pathway has not been well studied. Utilising transcriptomics, we sought to better understand how RIC confers such a profound reduction in intestinal injury.

In order to address the key biological question of what is different in the intestinal tissue following conditioning that provides a protective effect, the most obvious analysis to carry out therefore is between animals that have undergone the IRI insult to the bowel (IRI only) and those that had conditioning prior to the injury (RIC + IRI). It is expected that this analysis will be informative but the tissue in question has undergone a significant physiological stress and damage and thus whether the RNA recovered gives meaningful data was not a certainty. Moreover, whilst the control group for the comparison has also undergone the same insult, this will inevitably have diverse and extensive effects on multiple biochemical pathways. The potential for a lot of noise here means that analysis of the effect of RIC without the IRI is also desirable. Conversely, this analysis alone may not be fully informative as it is at least conceivable that the protective processes (in terms of meaningful changes in gene expression) in the end organ are triggered both by the conditioning and the stimulus of the ischaemic insult. Hence the decision to focus on both a comparison between animals who underwent RIC vs controls and animals who underwent RIC prior to IRI vs IRI alone.

Differential expression was seen in 868 genes in the RIC vs SHAM group and 135 in the RIC + IRI vs IRI alone comparison. Genes that are differentially expressed in both groups are likely to be important. There are 25 genes that are differentially expressed in both groups (Table 2). Of these 25 genes, eight are known to have to be involved with functions likely to be important in the mechanisms of RIC. Table 4 shows these eight genes and the results from the differential expression analysis; specifically the fold change in the expression levels and the corrected *p*-values.

**Table 4:** Genes showing differential expression in both the RIC vs SHAM and RIC + IRI vs IRI groups.

Genes	logFC	logCPM	*P* value	FDR
CXCL1				
RIC + IRI	− 0.73581	8.295629	9.37E−06	0.016462
RIC	− 0.59385	7.581155	6.20E−05	0.014521
TIMP1				
RIC + IRI	− 1.08748	4.695321	9.35E−05	0.036933
RIC	0.584135	6.188187	0.00031	0.021415
Cd55				
RIC + IRI	− 0.56804	7.283793	1.78E−05	0.020411
RIC	0.487938	6.174565	9.45E−05	0.016591
Map3k8				
RIC + IRI	− 0.75824	4.211773	0.000186	0.042433
RIC	− 2.7615	2.360486	0.000722	0.030387
Sod2				
RIC + IRI	− 0.98012	7.372327	0.000217	0.043591
RIC	− 1.47528	3.472257	0.000946	0.033794
Nfkb2				
RIC + IRI	− 0.49015	4.366177	0.000222	0.043961
RIC	− 0.3912	4.601592	0.000969	0.034104
Procr				
RIC + IRI	− 1.34504	0.765029	0.000227	0.044383
RIC	− 0.40365	7.534098	0.000969	0.034104
Usp36				
RIC + IRI	− 1.60342	− 1.88456	0.000267	0.045566
RIC	− 0.80179	2.778097	0.001428	0.040053

Genes that are differentially expressed in both comparisons, remote ischaemic conditioning (RIC) vs SHAM surgery (SHAM) and RIC plus ischaemic reperfusion injury (IRI) vs IRI.

*SHAM* Underwent fake surgery; *RIC* Remote ischaemic conditioning; *IRI* Ischaemic reperfusion injury; *IRI* + *RIC* Ischaemic reperfusion injury plus remote ischaemic conditioning; *NEC* Necrotising enterocolitis; *logFC* log fold-change in gene expression, *logCPM* log Counts per Million, *PValue* raw *p*-value, *FDR* False discovery rate (*p*-value corrected for multiple comparisons).

C-X-C Motif Chemokine ligand-1 (Cxcl-1) is a neutrophil chemoattractant^10^ that is downregulated by RIC. A Pubmed search showed no previous reports of a role of Cxcl-1 in the mechanisms of RIC. Interestingly, it has been established as an important part of the pathophysiology of brain damage after stroke. The production of Cxcl-1, through the NF-ĸβ-1 dependent pathway triggers neutrophil infiltration and thus leads to neuroinflammation^11^. Similarly, Cxcl-1 has been shown to recruit neutrophils in cardiac ischaemia^12^. Reduced expression of Cxcl-1 due to RIC, leading to less neutrophil recruitment in the presence of IRI, leading to less inflammation is a very plausible mechanism. Thus the effect of RIC maybe to reduce neutrophil mediated tissue damage.

Mitogen-Activated Protein Kinase Kinase Kinase 8 (MAP3k8), as with many of the genes discussed here, has an important role in oncogenesis. Map3k8 also has some interesting downstream effects. It induces the production of NF-ĸβ as well as the production of TNF-α and IL-2^10^. NF-ĸβ is discussed below but this shows consistent changes in expression at three different stages in the pathway; MAP3k8 induces NF-ĸβ and one of the downstream effects of NF-ĸβ is to increases the expression of Cxcl-1. In these data the expression of all three is suppressed.

Nuclear factor kappa-light-chain-enhancer of activated B cells pathway subunit 2 (NF-ĸβ2) is part of the NFĸβ family of transcription factors composed of five structurally related members including NF-ĸβ1 and NF-ĸβ2^13^. Functional NF-ĸβ2 is produced by the post-translational processing of its precursor protein p100. This may be especially important in the so-called ‘non-canonical’ NF-ĸβ pathway^14^. The precursor protein (p100) has an inhibitory function on NF-ĸβ^13^ and thus changes in the RNA expression levels could have promoter or inhibitory effects on the whole pathway, depending on whether the p100 protein is rapidly converted to mature NF-ĸβ2 or not. However, the Map3k8 and Cxcl-1 expression levels along with NF-ĸβ2 are all reduced in RIC suggesting that the overall effect is to inhibit NF-ĸβ pathway.

Superoxide Demutase 2 (SOD2) is an important part of the cell's response to oxidative stress and dismutates superoxide to hydrogen peroxide^15^. Hence, the reduced expression of SOD2 by RIC is at first glance surprising. However, increased production of hydrogen peroxide through the up-regulation of SOD2 stimulates pro-oxidants involved in apoptosis. Thus reduced expression of SOD2 can be said to be anti-apoptotic^16^.

Protein C Receptor (Procr) is a receptor for activated Protein C known as endothelial cell protein C receptor (EPCR)^10^. Activated Protein C is an important inhibitor of the clotting cascade but it is also exerts important anti-inflammatory effects^17^. The Protein C Receptor protein is found on the surface of endothelial and other cells types. Down-regulation of this receptor (seen in both RIC and RIC and IRI groups) implies a dampening down of Activated-Protein C suppression of coagulation. Coagulation is a cardinal feature of ischaemic necrosis^18^. Activated-protein C, via the Protein C receptor, triggers anti-inflammatory downstream effects via several mechanisms including suppressing NF-ĸβ^17,19^. Therefore, the down-regulation of this receptor in these data is potentially surprising, however it suggests other mechanisms are capable of reducing NF-ĸβ activation in this complex system, so lowering EPRC may be beneficial in this context. In addition, thrombin activation or EPCR produces a pro-inflammatory response and a disruption of the endothelial barrier^19,20^. Hence, EPCR has a pro-inflammtory mechanisms quite apart from the binding ligand that it is named for and reduced expression of this molecule would be expected to result in reduced inflammation. Although the cross-talk for this multi-ligand receptor is undoubtedly complex^14^.

Ubiquitin Specific Protease 36 (Usp36) could be a putative part of the RIC protective pathway because of its suggested role in autophagy^10^. Autophagy, a mechanism by which cells remove unnecessary or damaged components, is thought to be important in maintaining cellular function in response to stress^21^. Loss of Usp36 function autonomously activates autophagy^22^. This implies that reduced expression of Usp36 would cause the cell to increase its autophagic activity which could be important in responding to the severe stress of IRI. Conversely, work in the mouse and human kidney suggests that Usp36 interacts with SOD2 in the mechanism of acute kidney injury due to ischaemia. These data suggests that increased Usp36 expression would be protective^23^.

TIMP metallopeptidase inhibitor 1 (TIMP1) appears to have multiple functions. One such function is that it may be anti-apoptotic^10^. In these data, its expression is increased in RIC (compared to SHAM) but decreased in animals that undergo RIC and IRI. In the brains of rats, TIMP1 has been shown to be increased in response to infarction^24^. Thus the decreased expression in animals exposed to RIC and IRI compared to IRI alone may be a reflection of the reduced injury. However, the increase seen in RIC alone is less easily explained. As the name implies, TIMP proteins are named for their inhibition of metalloprotinases (MMPs) which in turn play a key role in the normal physiology and wound healing of connective tissue^25^. This inhibitory relationship is anti-inflammatory.

CD55 is a regulator molecule of the complement cascade^10^. Several studies looking at renal IRI have shown that over-expression of CD55 is protective against inflammation^26^. Although a specific role with respect to RIC has not been established. As with TIMP1, these results show increased expression of CD55 in the presence of RIC but in animals that underwent IRI and RIC, the expression is reduced compared to IRI alone. Thus if CD55 is part of the protective effect of RIC (and it clearly has an anti-inflammatory role in other contexts), its modulation of inflammation (presumably via the complement cascade) is not straight-forward.

Nuclear factor kappa-light-chain-enhancer of activated B cells pathway (NF-ĸβ), discovered in 1986^27^, has been extensively studied as a master regulator of pro-inflammatory genes^13^. Changes in the levels of TNF-α, IL-6 and IL-8 equivalent (KC/GRO) are seen in these animals. CXCL-1 showed reduced expression in both groups, indicating a concomitant down-stream effect of NF-ĸβ. Further corroboration is needed to confirm this but these data implicate a key role for NF-ĸβ in the protective effect of RIC in the intestine.

Toll-like Receptor-4 (TLR-4) triggers the NF-ĸβ, pathway^28^. Importantly, this is an early step in the pathogenesis of NEC, prior to the development of IRI. Indeed proponents of an immunological understanding of NEC, often cite the role of TLR-4 as being central to the development of the disease^29,30^. Ultimately, IRI is the common end-point of multiple pathways in NEC (although, it also leads to a vicious cycle of further inflammation and bowel compromise) thus one might expect RIC to only be effective at this late stage in the pathogenesis. Clearly, if RIC is effective in the intestine, it would be effective at reducing the ischaemic injury but if RIC is acting on NF-ĸβ as these data would suggest, then it is also likely to offer a protective effect at every stage in the pathogenesis of NEC. Whilst this hypothesis is not formally proven, it does present the prospect that RIC will be protective at multiple stages in the pathogenesis of NEC and thus (assuming that RIC can be delivered safely to human infants) could be a very effective therapy.

Five cellular markers were used for macrophages: CD163, CD206 and CD11c showed changes that were statistically significant, whilst the others (CD163 and CD68) showed no change. CD163 was increased by RIC, whilst CD206 and CD11c were reduced (Fig. S5). These different results suggest potentially that the cell population is not changing, rather the function of the macrophages is being altered. The regulation of these two proteins is different: CD163 is increased by IL-10, whilst CD206 is upregulated by IL-4 and IL-13^31^.

In addition, T-cell markers show an interesting pattern (Fig. 3). CD4 and CD8a were unchanged. CD3g was reduced by RIC. There was a non-significant trend suggesting an increase with IRI. Animals exposed to IRI showed a reduction compared to IRI alone. One way to understand this is that there is an increase in T-cells due to IRI which is mitigated/prevented by RIC. Given that the CD4 and CD8a markers are unchanged this change would have to be due to neither T-Helper or cytotoxic T cells. The pattern of CD69 expression shows a statistical increase in animals exposed to IRI with non-significant trends from RIC. Taking these two results together would suggest that it might be regulatory T-cells that are important here. CD69 is also expressed by NK-cells but the other NK-cell marker used, CD56 showed the opposite effect with RIC increasing the expression and KIR showing no change. Studies looking at the protective effect of RIC in the context of stroke^32^ and heart disease^33^ suggest a role for the spleen here, altering the population of circulation T-cells and their infiltration into tissue exposed to ischaemia. Intriguingly this may be part of the neural pathway of RIC as one study showed that denervation of the spleen had the same effect as splenectomy^33^.

Biological processes involving the role of HIF-1 in ischaemia is well-established. HIF-1 is a transcription factor made of two subunits (HIF-1α and HIF-1β) and is a key part of the cell’s response to hypoxia. Hence it is a marker of hypoxic insult at the cellular level. Under normoxic conditions HIF-1α has very high turnover, being constitutively expressed and broken down by proteasomal degradation. Hypoxia inhibits the breakdown of HIF-1α thus leading to its nuclear accumulation^34^. It has been shown that HIF-1α is increased in the intestinal injury of NEC^35^, which is part of the evidence base supporting the notion that NEC is an ischaemia–reperfusion disease. In these animal experiments we demonstrated that HIF-1α expression seems to be following the pattern of the intestinal injury and this is mitigated by RIC: HIF-1α is increased with IRI and this effect it mitigated with RIC. HIF-1β is unchanged, whist HIF2α and VEGFα follow the same pattern as HIF-1α (Fig. 12). Given the way that HIF-1 functions with constitutional expression of HIF-1β, it is logical that there is a change in the expression of HIF-1 α and not HIF-1β. It is however perhaps surprising that there is a change at all when it is known that HIF-1 is regulated at the post-translational level. Thus these data are suggesting that RIC is having an effect at the transcriptional level. HIF-2 has a similar function to HIF-1 in endothelial cell responses to hypoxia. There is a switch from HIF-1 to HIF-2 which is part of the cellular adaption to hypoxic stress^36^. VEGFα is major marker of hypoxic stress, regulated by HIF^37^.

These data together show how RIC is mitigating the hypoxic stress in the tissues. Whether this is part of the protective mechanism of RIC or simply a marker of the reduced injury because of RIC is not clear but it is certainly plausible that HIF and VEGF are part of a pathway by which RIC is protecting the tissue. Similarly, The cytokeratines examined KRT-7, -8, and -18 all showed the same pattern of an increase with IRI which was mitigated by RIC (Fig. S7). This suggests that either they are playing a role in the pathology of IRI that is reduced by RIC leading to less injury or conversely that they are a marker of the severity and requirement for healing of the injury. In this context fibroblasts and smooth muscle markers were increased by IRI with the effect mitigated by RIC (Fig. S6). These cells are involved in wound repair and are required to support tissue healing and homestasis to maintain the epithelail barrier and thus maybe protective in NEC pathophysiology.

Toll-like receptor 4 (TLR4) is thought to be a key part of the mechanism. The Toll-like receptors are part of the innate immune system that trigger an inflammatory response to bacterial infection by recognising common epitomes that are highly conserved in bacterial species. TLR4 activation triggers increased expression of MyD88 and NF-ĸβ. These data show that TLR4 expression is decreased by IRI. However, the down-stream effects of increased MyD88 and NF-ĸβ are seen in this model. Moreover, both of these genes show reduced expression in response to RIC. In the case of MyD88, there is a mitigation of the effect of IRI therefore the reduction might not be realised if something other than IRI was triggering the rise. However, NF-ĸβ expression is suppressed independent of IRI.

This supports the concept that the model is a reasonable representation of the human disease and that there is some cross-over in the pathways such that RIC could have a protective effect against NEC both in terms of reducing the necrosis but also in terms of interrupting the pathophysiological pathways earlier. Whilst RIC has of course been investigated primarily as a potential therapy for specifically ischaemic injury, more recent work has looked at the anti-inflammatory potential^38^. Because these pathways interact and overlap, there is good reason to think that RIC may have a beneficial effect even in the earlier stages of NEC before necrosis develops.

The reperfusion injury salvage kinase pathway (RISK), in the context of cardiac ischaemia–reperfusion is an important part of the protective cellular mechanism. We hypothesise that there is an equivalent pathway in the intestine. Genes in this pathway include MEK1, MEK2, and PI3K. In each case the expression of these genes is decreased by IRI. With MEK1, the expression is unchanged by RIC. With MEK2, RIC decreases the expression but not be as much as IRI does. In the case of PI3K, RIC increases the expression. The RISK pathway is an anti-apoptotic cascade and works by inhibiting the opening of the mitochondrial permeability transition pore (mPTP)^39^. Therefore it is not surprising that in the context of widespread ischaemia all the proteins in the pathways show reduced expression. Equally, because it is a cascade (primarily mediated by phosphorylation), it is also not surprising that there is no clear change in expression seen at the RNA level. A cascade like this works by (in this case) phosphorylation of proteins. Thus a change in the expression level of just one of the proteins involved with have a knock-on effect on all the down-stream proteins.

The increase in expression PI3K, due to RIC, is seen in both the animals exposed to IRI and those that were not, suggesting a potential key role here for this molecule in the protective mechanism of RIC in relation to the RISK pathway. These data show that RIC increases this expression and that if IRI occurs, expression, whilst much lower than baseline is still higher than that seen in IRI alone. IRI results in a decrease in expression but this decrease is mitigated by RIC. Phosphoinositide 3-kinases are a family of signal transducer enzymes that function by phosphorylating the 3 position hydroxyl group of the inositol ring of phosphatidylinositol^40^. Hausenloy et al*.* (2012) studied the role of P13K in RIC in the porcine heart^41^. Their data showed that the protective effect of RIC on the heart could be abolished by administration of a blocking agent of PI3K (Wortmannin). The role of PI3K was also confirmed by Western-blotting analysis. The increase in PI3K expression in the intestine demonstrated here would be consistent with an equivalent RISK pathway existing in the intestine. In these data, PI3K increased by RIC (RIC vs SHAM: *p* = 0.023; RIC + IRI vs IRI: *p* < 0.001), Table 3, Fig. 6.

P38 and Protein Kinase C were studied by Heinen et al*.* (2011)^42^ with Western Blotting analysis to study the protective pathways of ischaemic conditioning – both direct and remote. Their data showed a role for P38 and PKC in direct ischaemic conditioning but in their data in the heart, the expression of P38 and PKC did not change with *remote* conditioning^42^. These data are consistent with those results as both P38 (IRI vs SHAM: *p* = 0.001; RIC + IRI vs RIC; *p* = 0.012) and PKC (IRI vs SHAM: *p* < 0.001; RIC + IRI vs RIC, *p* = 0.01) in the intestine show altered expression with IRI but not with RIC. Table 3, Fig. 6.

It is intriguing that these pathways clearly play a role is the protective effect of conditioning when the conditioning is applied directly to the target organ but seem to have little/no role in the same protective effect when the conditioning is delivered remotely. However, these results are entirely consistent with these findings in an entirely different organ system. Further supporting the hypothesis that the mechanisms in the intestine are similar to that that seen in cardiac tissue.

No previous studies have been published examining the transcriptome of the intestine in response to RIC. Multiple pathways of NEC pathogenesis have been proposed. No animal model completely replicates the human disease and rats (used in this work) are no exception. The importance of bacterial colonisation and invasion in the human disease is certainly not fully replicated in this (or any other) model. However, there are immune pathways that are important in NEC before it gets to the stage of necrosis. This model best mimics the common end-point of NEC with a pattern of bowel necrosis and systemic effects very akin to the human disease. The mechanisms by which RIC is protective may also function at this level—by driving an anti-inflammatory response as well as the resistance to ischaemia; if this is true then RIC would potentially provide a protective effect against NEC earlier in the pathophysiology.

In conclusion, the data shown here suggest several promising avenues for research. The changes in gene expression seen by comparing IRI with the SHAM group show that this model, as well as generating a macroscopic injury that is similar to severe NEC^4,43,44^, mimics many known biochemical pathophysiological pathways. There is evidence that an intestinal equivalent of the RISK pathway in cardiac tissue may exist, with data showing significant increases in PI3K with RIC and RIC/IRI and that RIC is triggering an anti-inflammatory process, involving known pathways of the innate immune system, including NFKB, a very important regulator of inflammation.

## Methods

### Animal model

Animal experiments were carried out with ethical approval from the local Animal Welfare and Ethical Review Body (University of Southampton)^4,43,44^. All experiments were carried out subject to the relevant UK law (Animals in Scientific Procedures Act (APSA) 1986 and revisions^45^ (Project licence: PA813F125)). This study is reported in accordance with the ARRIVE guidelines^46^.

Intestinal injury was induced in suckling rat pups aged 10–13 days, under terminal (isoflurane) anaesthesia. Laparotomy was performed and IRI induced by occlusion of the SMA for 40 min with a microvascular clip, followed by 90 min of reperfusion.Controls underwent SHAM surgery. RIC was induced by means of a ligature applied to the hind limb to occlude arterial inflow. Each animal undergoing RIC received 3 cycles of 5 min of ischaemia with 5 min of reperfusion between the ischaemia episodes. The animals were randomly allocated to four groups: SHAM; RIC; IRI; RIC + IRI (Fig. 6). The protocol is describes more fully in our previous publication^4^.

### RNA isolation and preparation

At the end of the procedure, 2 cm of ileum (3–5 cm from the ileo-caecal valve) was removed for quantitative RNA analysis. This was immediately flushed with Phosphate Buffered Saline (with calcium chloride and magnesium chloride.) (Gibco/Thermo Fisher Scientific) and then placed in RNAlater™ Stabilization Solution (Qiagen) and stored at −20 °C, as per the manufacturer’s instructions. The RNA extraction, library preparation and next-generation sequencing were performed by Qiagen Genomic Services (Hilden, Germany). Alignment of the mRNA reads to the genome was also performed by Qiagen, who provided the raw-count matrix used for analysis of differential expression. The workflow is shown in Fig. S8.

RNA Extraction and quality control was performed using the RNeasy Plus protocol which uses phenol/guanidine for lysis and silica-membrane purification of RNA. Sample quality control was then performed on each sample. The quantity of RNA was determined spectrophotometrically by measuring the absorbance at 260 nm. The integrity of the total RNA purified was then assayed with the Agilent 4200 TapeStation system (Agilent Technologies Ltd, USA). 2 μl of each sample was used to assess RNA purity generating an RNA Integrity Number (RIN). RIN scores above 8 are considered ‘high quality;’ scores of 5–7 indicate some degree of fragmentation^47,48^.

### Library preparation

The library was prepared using the TruSeq® Stranded mRNA Sample preparation kit (Illumina Inc) which produces a cDNA library for whole transcriptome analysis. Briefly, the starting material (500 ng) of total RNA was mRNA enriched using the oligodT bead system. The isolated mRNA was subsequently fragmented using enzymatic fragmentation. Then first strand synthesis and second strand synthesis were performed and the double stranded cDNA was purified (AMPure XP, Beckman Coulter). The cDNA was end repaired, 3’ adenylated and Illumina sequencing adaptors ligated onto the fragments ends, and the library was purified (AMPure XP). The mRNA stranded libraries were pre-amplified with PCR and purified (AMPure XP). The libraries size distribution was validated and quality inspected on a Bioanalyzer 2100 or BioAnalyzer 4200 TapeStation (Agilent Technologies). High quality libraries were pooled in equimolar concentrations based on the Bioanalyzer Smear Analysis tool (Agilent Technologies). The library pool(s) were quantified using qPCR and optimal concentration of the library pool used to generate the clusters on the surface of a flowcell before sequencing.

### Next-generation sequencing

The sequencing of the RNA was preformed using an Illumina NextSeq 550 High Throughput Next Generation Sequencer. (Qiagen, Germany/Illumina, USA). The transcriptome was sequenced using the following parameters: 30 M reads and a read depth of 75 base pairs.

### Transcriptomic alignment

The analysis stage where the transcriptomic reads were aligned to the genome was performed using CLC read mapper within Qiagen’s Biomedical Genomics Workbench software (Qiagen, Germany/CLC bio, Denmark)^49^. Benchmarking studies have shown this gives equivalent or better performance compared to widely used aligning tools^50^.

### Analysis

Analysis of the mapped counts was conducted using *R* v 3.6.1 (R Foundation for Statistical Computing, Vienna, Austria)^51^. The *R* Script used is included in the additional material. To test for potential confounders, known phenotypic data for each animal was entered. The age in days, gender, weight, from which litter the animal came and the date of the experiment was entered for each animal and linked to the corrosponding expression values. Before formal analysis of the data, the raw data was explored with principal component analysis (PCA) to perform quality control, with searchs for batch effects and removal of significant outliers from the model if necessary.

### Data exploration and quality control

Lowly expressed mRNA genes were excluded in order to reduce the need for multiple testing correction and thus reducing the risk of a Type II error and inconsistent reads across samples. Using the whole dataset; Hierarchical clustering was performed using the Euclidean distance method; PCA was performed and plotted; and median vs interquartile range for each raw count was plotted. Finally the PCA was plotted with the samples labelled for the known phenotypical factors. Any sample that was shown to be an outlier in more than one of these plots would be considered as a true outlier and excluded from further analysis.

### Differential expression testing

The differential gene expression was assessed in four 2-way comparisons, using edgeR (version 3.6.1)^11,12^.

### Differential expression: targeted gene analysis

As well as performing this undirected differential expression testing, we interrogated the data for specific genes of interest. These broke down to various sub-groups: markers of cell types (Table 5), markers of biological processes (Table 6), proteins known to be important in the pathogenesis of NEC (Table 7) and proteins that are found in known biological pathways of RIC in other tissues (Table 8). In order to compare these different genes, simple box-plots and t-tests were performed using R.

**Table 5:** Selected proteins used as markers of the cell populations.

Protein name	Gene name	Marker for
CD45	PTPRC	Immune cells
CD163	CD163	Macrophages
CD164	CD164	Macrophages
CD206	MRC1	Macrophages
CD68	CD68	Macrophages
CD11c	ITGAX	Macrophages
CD20	MS4A1	B Cells
CD19	CD19	B Cells
CD3	CD3G	T Cells
CD4	CD4	T Cells
CD8	CD8A	T Cells
CD103	ITGAE	T Cells
CD69	CD69	T Cells
CD56	NCAM1	NK Cells
KIR	KIR3DL1	NK Cells
CD109	CD109	Endothelial Cells
CD31	PECAM1	Monocytes
CD15	FUT4	Neutrophils
Vimentin	VIM	Fibroblasts
N-cadherin	N-cadherin	Fibroblasts
CDH2	CDH2	Fibroblasts
Desmin	DES	Alpha smooth muscle
CDH2	CDH2	Alpha smooth muscle

This table details the specific gene markers used within this study. The gene names refer to the names used by the Ensembl database^52^.

**Table 6:** Selected proteins used as markers of hypoxia and cellular injury.

Protein name	Gene name	Marker for
HIF1α	HIF1α	Hypoxia
HIF2α	ARNT	Hypoxia
HIF1β	EPAS1	Hypoxia
VEGF	VEGFA	Hypoxia
KRT7	KRT7	Type 1 cytokeratins—cell repair
KRT8	KRT8	Type 1 cytokeratins—cell repair
KRT18	KRT18	Type 2 cytokeratins—cell repair
KRT19	KRT19	Type 2 cytokeratins—cell repair

The gene names refer to the names used by the Ensembl database^52^.

**Table 7:** Selected proteins shown to be important in the pathophysiology of NEC.

Protein name	Gene name	Function
TLR-2	TLR-2	Toll-like receptors are part of the innate immune response to bacteria. Activation of TLRs in known to be important in NEC^53–55^
TLR-4	TLR-4	
MyD88	MyD88	Downstream signal from TLR4^56^
NF-kβ	NFKβ1	Cellular response to stress^56^
PAF	PTAFR	Platelet activation/proinflammatory cytokine^56^
CD17	KIT	Marker for regulatory T-cells^57^
iFABP	iFABP	Putative biomarker of NEC^58^

The gene names refer to the names used by the Ensembl database^52^. References cited are evidence for the role of these molecules in the pathogenesis of NEC.

**Table 8:** Selected proteins shown to be important in the mechanisms of RIC in other target organs.

Protein name	Gene name	Function
PKC	PRKCA	The Risk Pathway, shown to be important in the mechanisms of RIC in cardiac tissue^39^
P38	MAPK14	
PI3K	PIK3C3	
MEK 1	MAP2K1	
MEK 2	MAP2K2	

The gene names refer to the names used by the Ensembl database^52^.

### Supplementary Information

Supplementary Figures.

Supplementary Information 1.

## Data Availability

The datasets generated and/or analysed during the current study are available in the University of Southampton repository, https://eprints.soton.ac.uk/478709/4/Raw_Counts_All_Samples.csv. The raw sequence reads are available from the National Center for Biotechnology Information (NCBI) Bioproject database (Ascension number 1067150). https://www.ncbi.nlm.nih.gov/bioproject/1067150.
