# Supplementary material for: Transcriptomic analysis of the effect of remote ischaemic conditioning in an animal model of necrotising enterocolitis
Source: Sci Rep. 2024 May 11;14:10783. doi: 10.1038/s41598-024-61482-9 (PMC11088709; doi:10.1038/s41598-024-61482-9)
Supplement: Supplementary file 1 — Supplementary Figures. [file 41598_2024_61482_MOESM1_ESM.pdf]

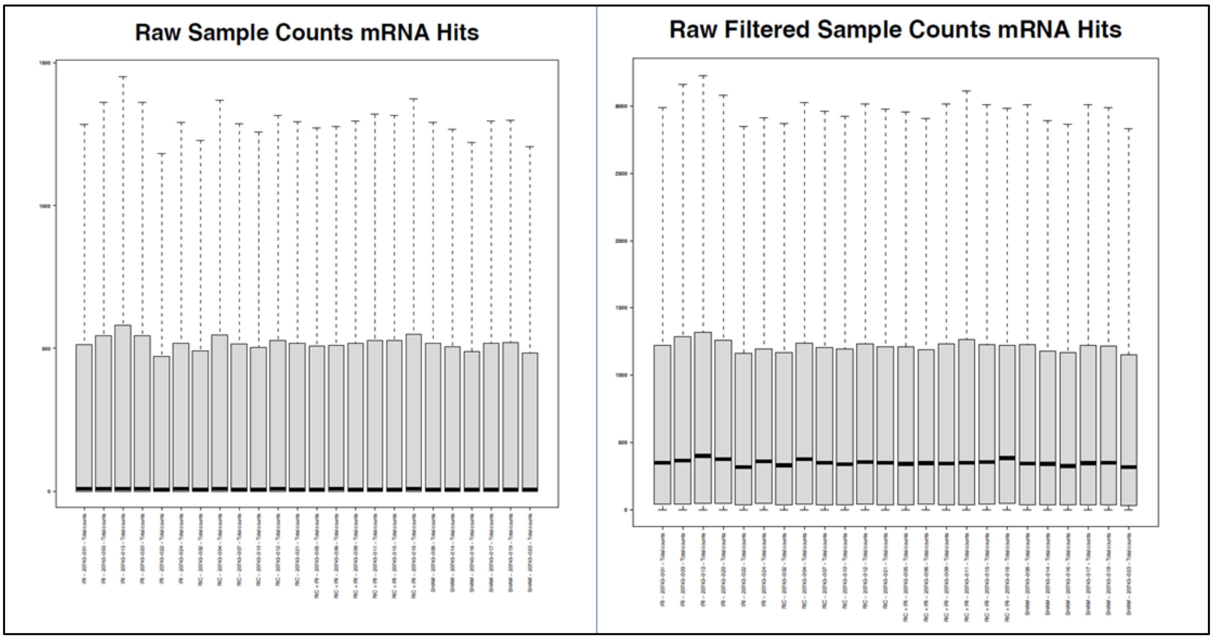

Figure S1 Box plot of Unfiltered and Filtered Sample Counts of mRNA Hits in each sample; The filter removes lowly expressed mRNAs. Median shown by black gene. Gene counts range from 0-1500 copies. Box shows 1.5x IQR. This is done to reduce noise and prevent Type II error.

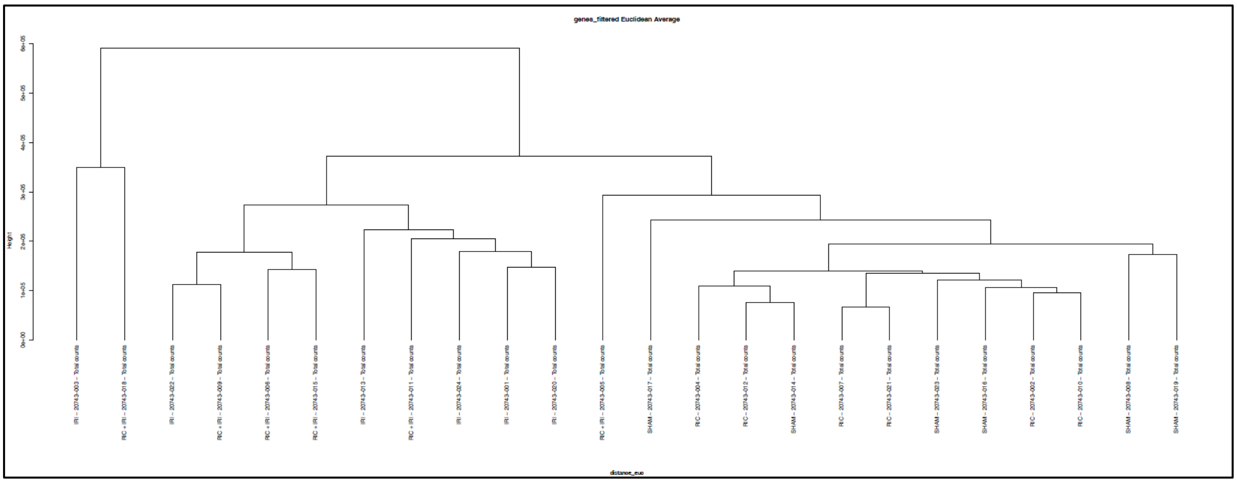

Figure S2 Hierarchical clustering of all samples from an RNA-Sequencing experiment which compared four conditions (list them). Using Euclidean distance and average linkage clustering showed that IRI vs No predominately defined the clusters in the whole model.

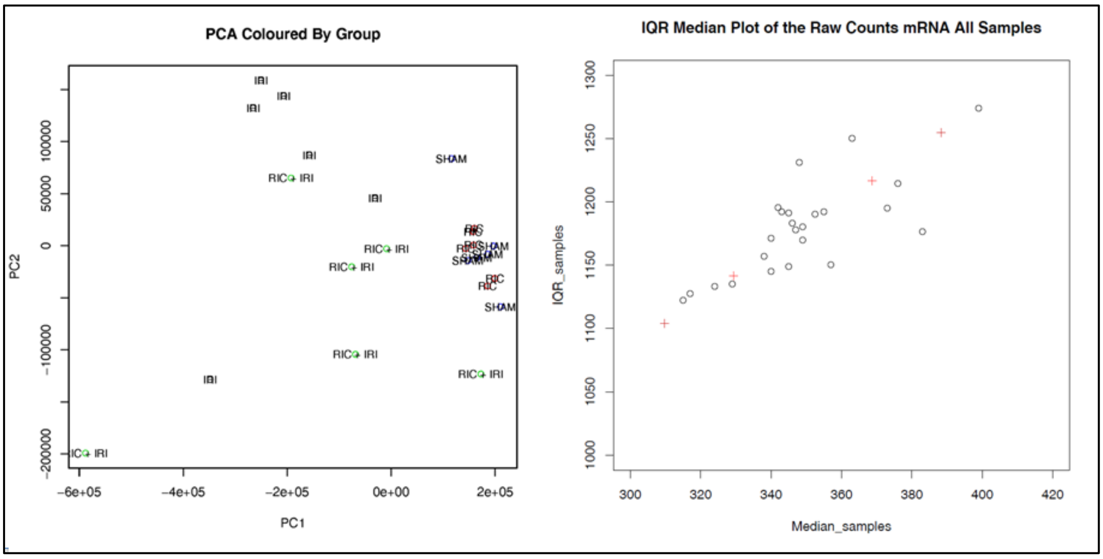

Figure S3 Principal component analysis (PCA) of all samples labelled by group. Median or Raw Counts plotted against Interquartile range of all samples and genes

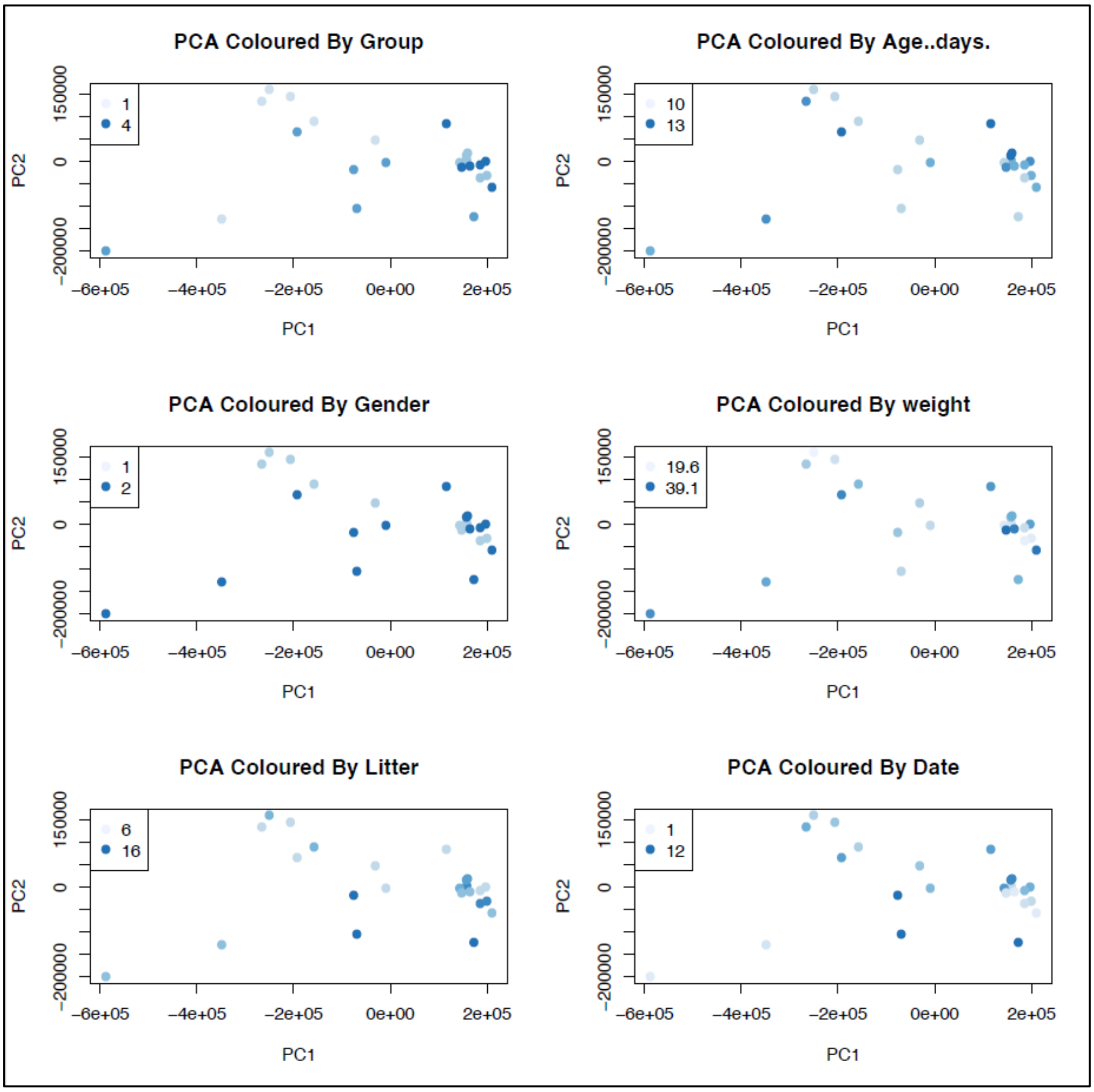

Figure S4 Principal component Analysis (PCA) of RNA Sequencing data of all 4 groups. These graphs showing that the known potential confounding factors are not showing a measurable effect. Factors examined: Age of pups; gender; weight; litter the pups were from; date of experiment.

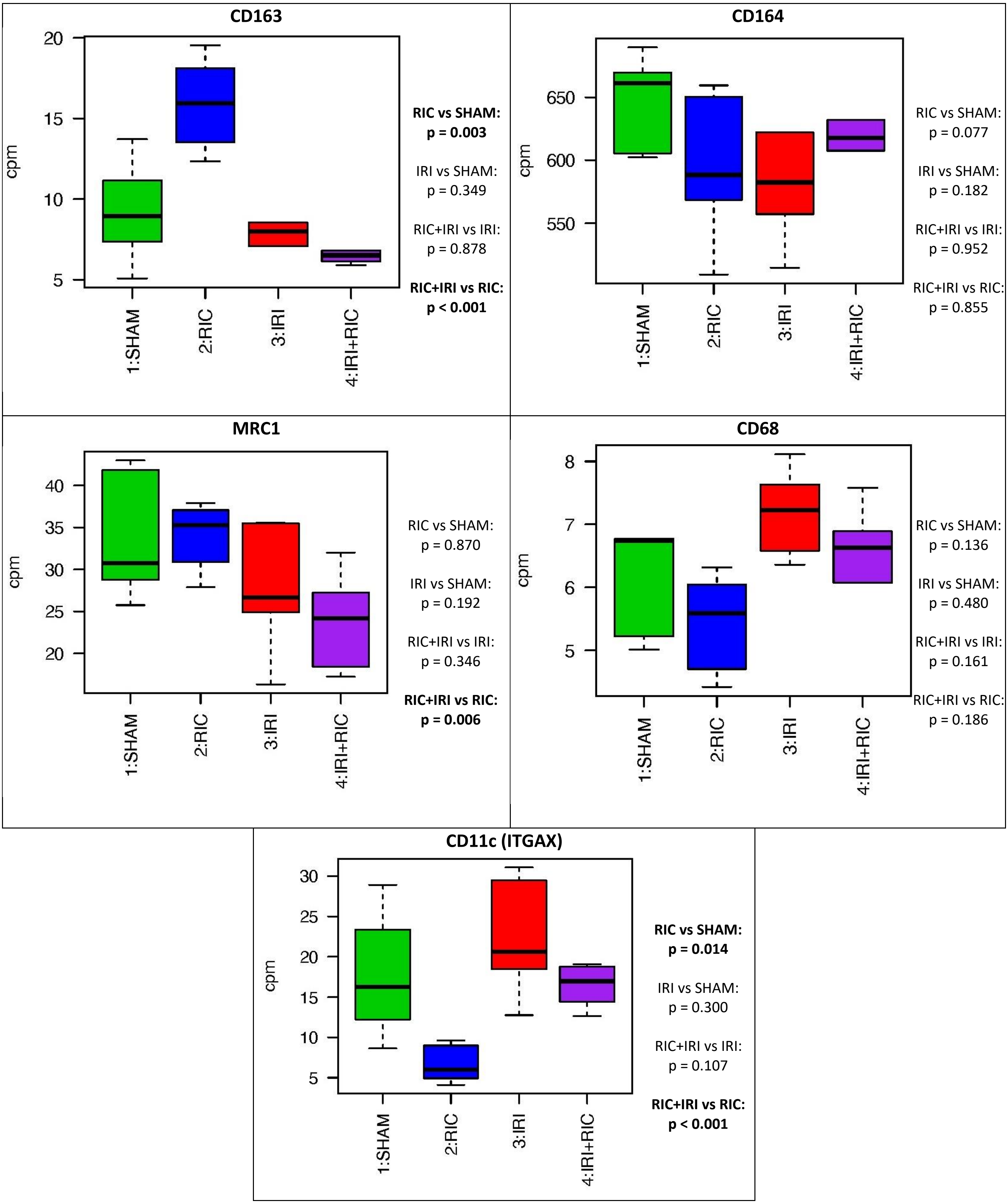

Figure S5: Four way comparison of gene expression of specific markers: Cell type markers for macrophages p-values derived by t-test comparison between the indicated groups

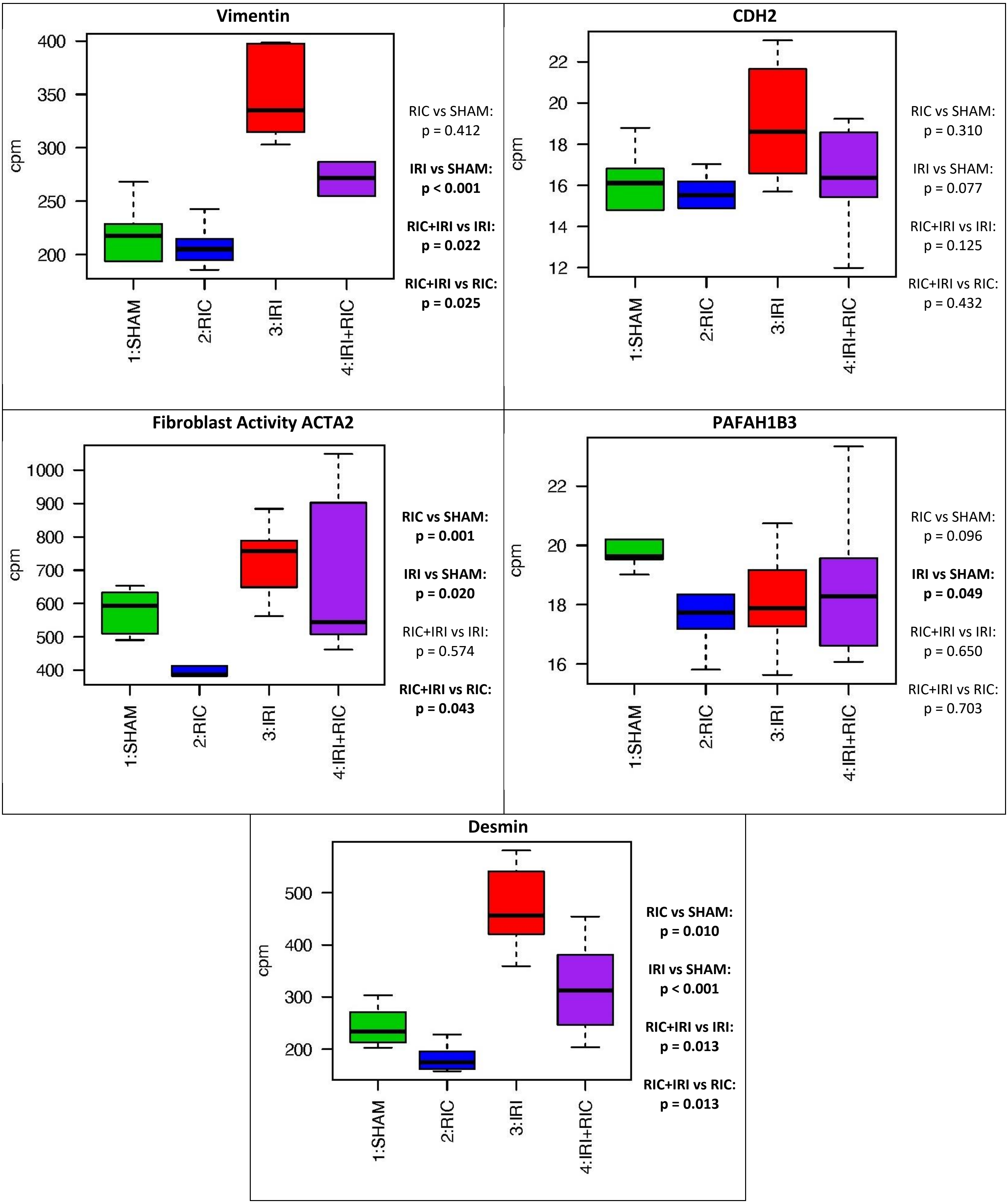

Figure S6: Four way comparison of gene expression of specific markers: Cell type markers for fibroblasts and alpha smooth muscle p-values derived by t-test comparison between the indicated groups

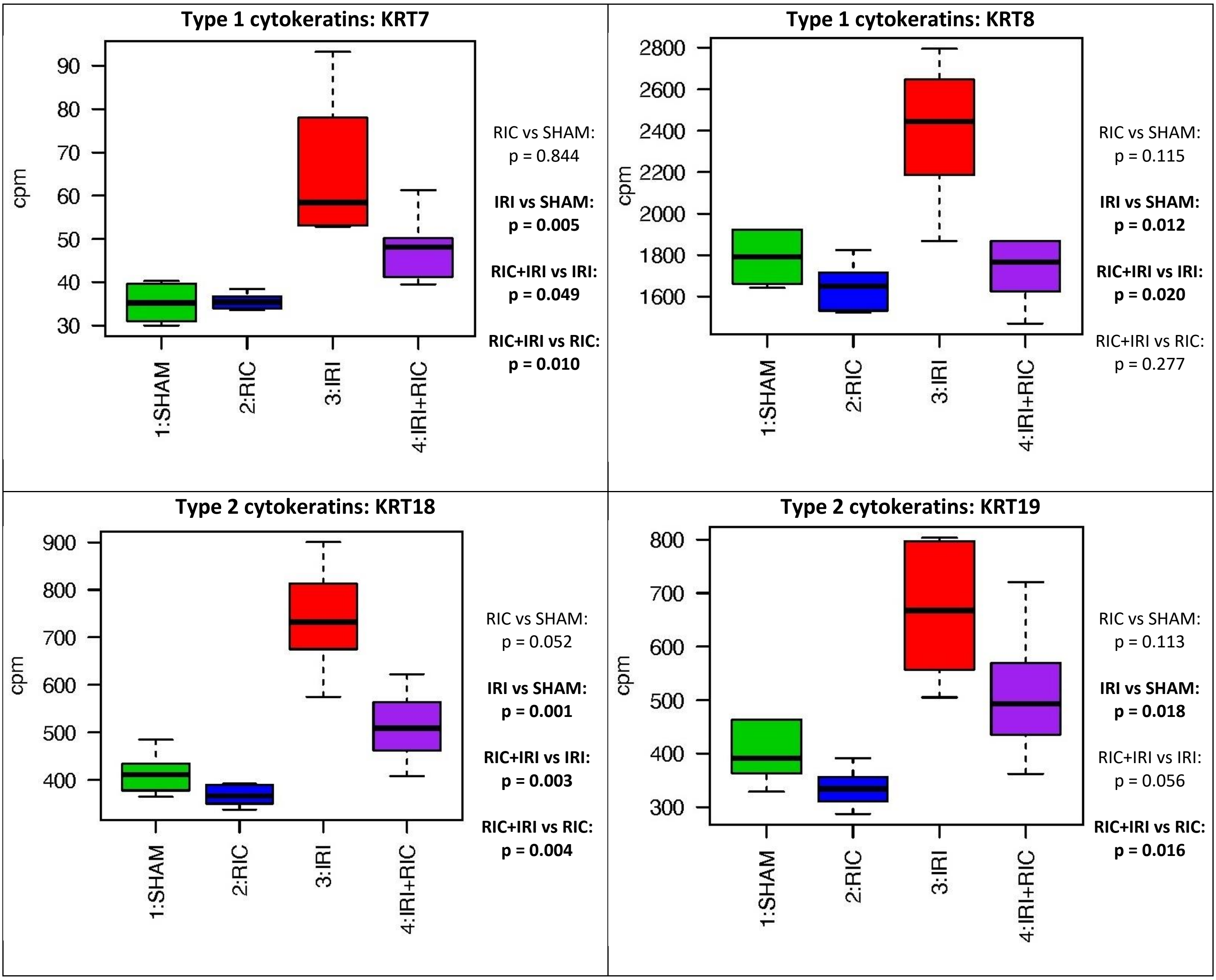

Figure S7: Four way comparison of gene expression of specific markers: Markers of cell toxicity p-values derived by t-test comparison between the indicated groups

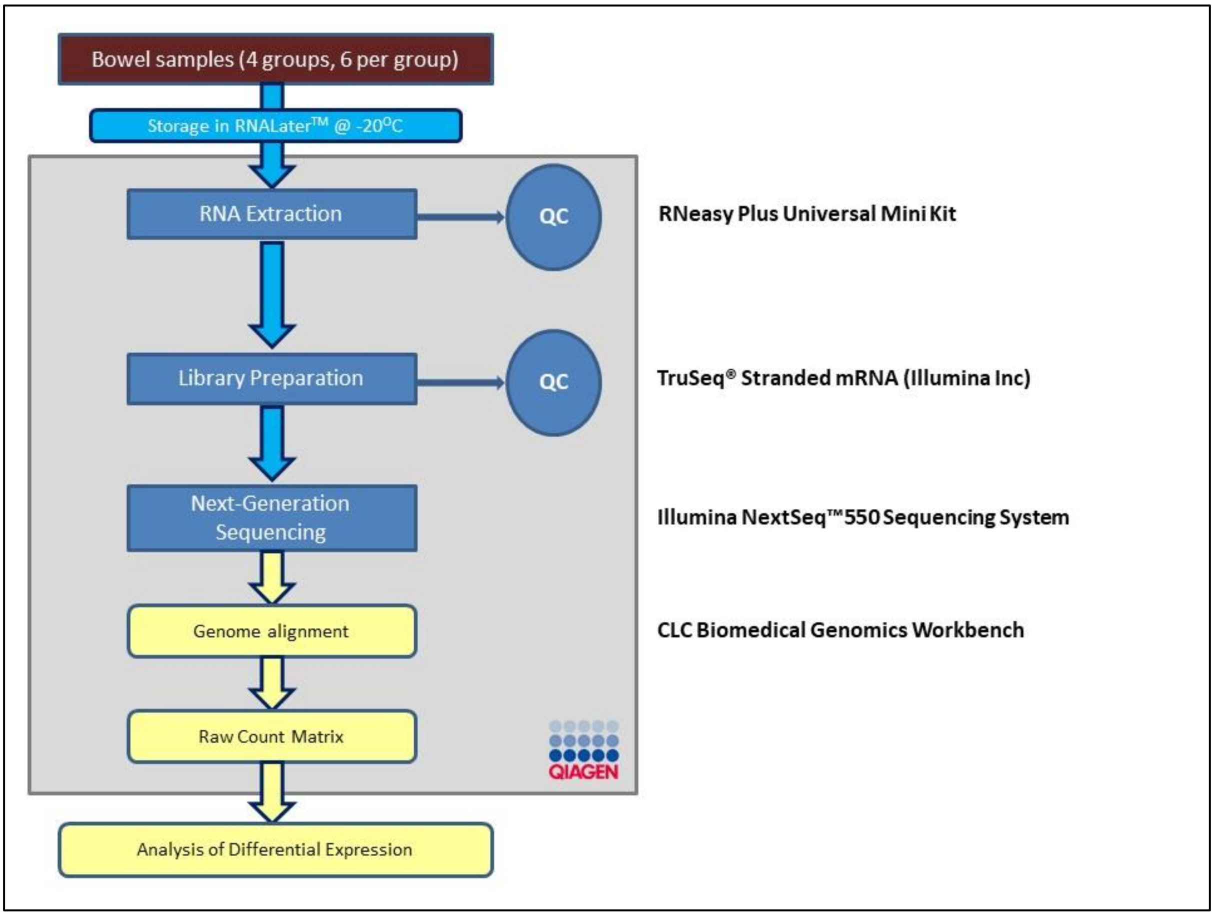

Figure S7 Experimental Protocol Flow Diagram: Samples of bowel tissue were sent to Qiagen in RNAlater™. All the steps in the grey box were performed by Qiagen Laboratories. The 'Wet-lab' work prior to RNA extraction is described in detail Jones *et al.* (2022).
